# Automatic Detection of Pectoral Muscle Region for Computer-Aided Diagnosis Using MIAS Mammograms

**DOI:** 10.1155/2016/5967580

**Published:** 2016-10-25

**Authors:** Woong Bae Yoon, Ji Eun Oh, Eun Young Chae, Hak Hee Kim, Soo Yeul Lee, Kwang Gi Kim

**Affiliations:** ^1^Biomedical Engineering Branch, Division of Convergence Technology, National Cancer Center, Goyang, Republic of Korea; ^2^Department of Radiology, College of Medicine, Ulsan University, Seoul, Republic of Korea; ^3^Electronics and Telecommunications Research Institute, Daejeon, Republic of Korea

## Abstract

The computer-aided detection (CAD) systems have been developed to help radiologists with the early detection of breast cancer. This system provides objective and accurate information to reduce the misdiagnosis of the disease. In mammography, the pectoral muscle region is used as an index to compare the symmetry between the left and right images in the mediolateral oblique (MLO) view. The pectoral muscle segmentation is necessary for the detection of microcalcification or mass because the pectoral muscle has a similar pixel intensity as that of lesions, which affects the results of automatic detection. In this study, the mammographic image analysis society database (MIAS, 322 cases) was used for detecting the pectoral muscle segmentation. The pectoral muscle was detected by using the morphological method and the random sample consensus (RANSAC) algorithm. We evaluated the detected pectoral muscle region and compared the manual segmentation with the automatic segmentation. The results showed 92.2% accuracy. We expect that the proposed method improves the detection accuracy of breast cancer lesions using a CAD system.

## 1. Introduction

Mammography is an important method for the diagnosis of breast diseases such as breast cancer. Studies on computer-aided detection (CAD) systems that help radiologists with the diagnosis of breast cancer are now being conducted actively. The CAD systems provide objective and more accurate information to reduce the misdiagnosis of breast cancer. In addition, the CAD systems enable radiologists to focus on the region of interest [1–3].

Mammogram images have different shapes when scanned from different angles. The pectoral muscle is shown in two different views: right mediolateral oblique (RMLO) and left mediolateral oblique (LMLO). Normally, the acquired images are divided into three different regions (breast boundary, background, and pectoral muscle) for the automatic detection of lesions in the CAD system. Through this process, the mammographic image transformed a data into meaningful information for further analysis [4, 5].

The pectoral muscle segmentation is necessary for the detection of microcalcifications or mass because the pectoral muscle has a similar pixel intensity as that of lesions, which affects the results of automatic detection. Besides, in mammography, the pectoral muscle region is utilized as an index to compare the symmetry between LMLO and RMLO views. Therefore, an algorithm for dividing the pectoral muscle is needed to detect microcalcifications or mass. Several researches have conducted to formulate a method for detecting the pectoral muscle [6–8].

Digital database for screening mammography (DDSM) and mammographic image analysis society (MIAS) database are used for setting the target, which is the basis for the measurement in image processing. These databases are also used for the comparison between studies such as the automatic detection of lesions. The intensity and contour-based method have been taking advantage in mammographic image processing utilizing the image databases.

Raba et al. used a region growing method for the detection of lesions utilizing the pixel intensity. In this method, 300 images from the MIAS database were tested. As a result, nearly 98% of the images were “correct” and 86% of the images were “good” [9]. Alam and Islam detected the pectoral muscle using *K*-means and a region growing method. This method was about 4% more accurate than the Raba et al. method (291/322, 90.3%) [10]. The average of false positive (FP) and false negative (FN) was 5% and 1%, respectively. The average of true positive (TP) was 94.4%. Kwok et al. obtained binary images using contour-based detection methods and fitting a line by extracting the outline information [11]. Camilus et al. segmented a mammographic image by watershed method. This method was based on the pixel intensity. It processed a total of 84 mammograms in the MIAS database. The mean FP and FN were 0.85% and 4.88% [12]. Kinoshita et al. detected the pectoral muscle using the canny edge method, which converts the images in the radon transform method. In this method, Kinoshita et al. used uncommon images (540 cases, not MIAS database) [13]. The images obtained from the manual detection were compared with that obtained from the automatic detection. The result was defined as “correct” (28.9%) when the differences in both the FP and FN were less than 5%. It was defined as “acceptable” (40.7%) when FP and FN were lower than 5% and 15%, respectively. It was defined as “unacceptable” (30.4%) when FP and FN were higher than 15%. Besides, several detection methods such as wavelet transform, Sobel edge detection, Hough transform, polynomial fitting, and random sample consensus (RANSAC) algorithm are widely used [14, 15].

In mammographic images, the pectoral muscle has an oblique outline. Generally, the features of the pectoral muscle region have a high intensity compared to the other tissues. In this study, we proposed a different type of detection method for improving the detection rate in the CAD system. The proposed method was improved to enhance the image contrast, and the outline was detected using the customized oblique kernel. The outline of the pectoral muscle was detected using a fitting method based on the extracted image following the RANSAC algorithm. In the proposed method, the pectoral muscle region was detected using a morphological method with pixel intensity. In order to supplement a limit for the method, the Hough transform method was used. The MIAS database was used for evaluation. This method compares the automatically and manually drawn regions for obtaining the pectoral muscle. The results were more accurate in specific cases.

## 2. Materials and Methods

**Figure 1:**
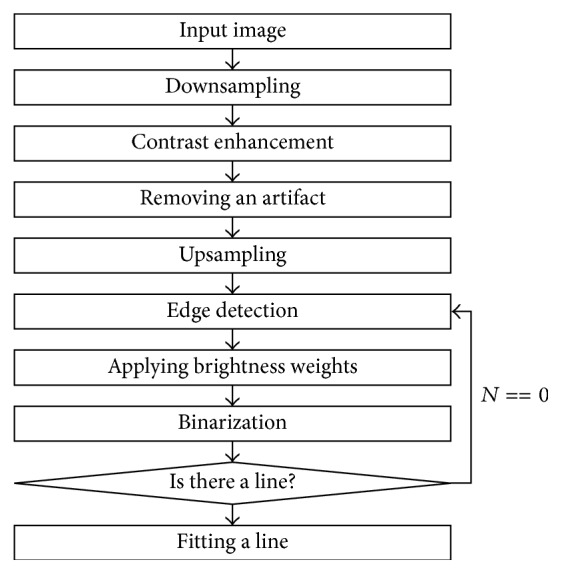
A flowchart for detecting the pectoral muscle.

The pectoral muscle has an oblique outline similar to diagonal. The pixel intensity of the pectoral muscle region is high compared with the other regions. The proposed method consists of three stage, preprocessing, segmentation, and postprocessing (Figure 1). In the preprocessing stage, the breast region and the background region are divided by the binarization with a threshold value, which is obtained from the histogram of the image.

### 2.1. Image Data

The proposed method used images from the Mini-MIAS database. MIAS is a research organization in the UK that studies mammography. This institution holds the mammogram data. The database has 322 cases with an image resolution of 1024 × 1024 pixels. Scanned film mammographic images reduce the resolution from 50 *μ*m to a maximum of 200 *μ*m. Each case is composed of a single image without the knowledge of the four views (LMLO, RMLO, LCC, and RCC).

### 2.2. Preprocessing

#### 2.2.1. Removing Artifact

**Figure 2:**
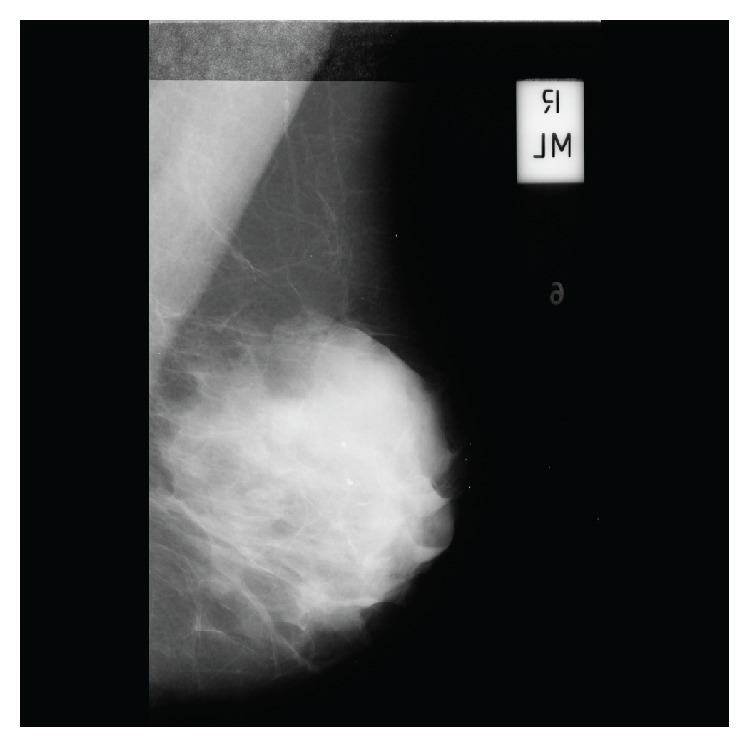
Mammographic image in the MIAS database.

In general, MIAS images have inconsistent margins in the left and right edges (Figure 2). This is because of the digitized film mammography. Therefore, it needs preprocessing. A line-profile method was used to remove the blank space in the preprocessing image. This approach sums the pixel values corresponding to each pixel column and finds the two rapidly changing points. It removes the blank space that does not correspond to the two points. Some images have many artifacts. The proposed method used a labeling algorithm for removing the artifacts. The artifacts were removed except the largest region after the labeling process.

#### 2.2.2. Contrast Enhancement

**Figure 3:**
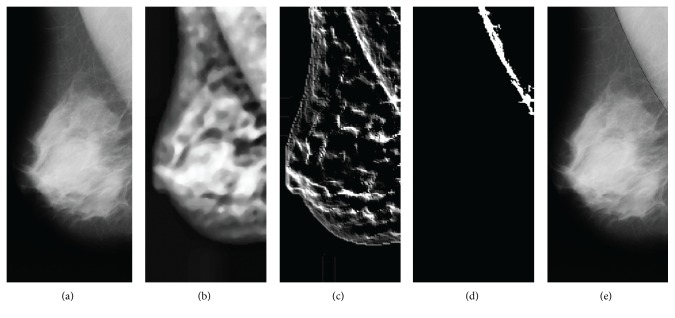
Mammographic image processing, (a) removing blank space, (b) image enhancement, (c) edge detection, (d) selecting the candidate line, and (e) result of pectoral muscle region detection.

Figure 3(a) is an image obtained by windowing the maximum and minimum pixel value within the breast region. The contrast enhancement of the pectoral muscle outline is applied to the contrast-limited adaptive histogram equalization (CLAHE) filter, as shown in Figure 3(b), and the noise is removed with a median filter. The CLAHE filter in the image contrast enhancement method merges the results of the image smoothed with bilinear interpolation divided by the number of blocks. Each block is carried out independently of the histogram equalization.

### 2.3. Outline Candidate Detection of the Pectoral Muscle

#### 2.3.1. Outline Image Reconstruction according to the Pixel Position

**Figure 4:**
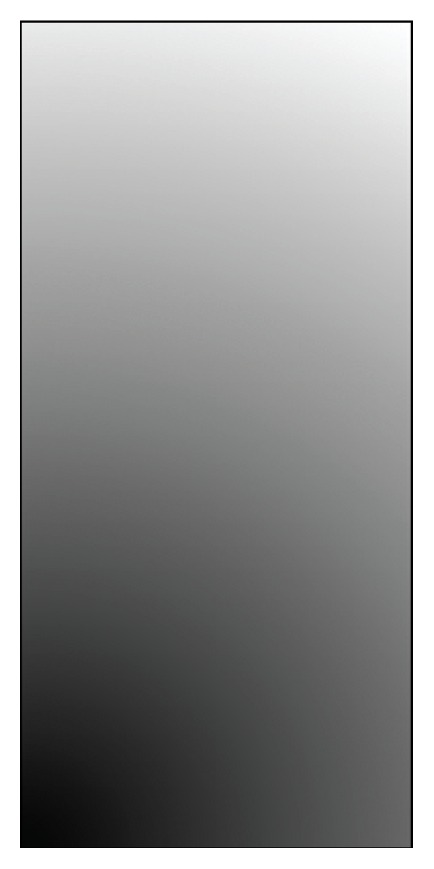
Linear-wedge gray scale image.

The outline component was detected using the oblique kernel in Figure 3(c). The kernel was used from the upper left toward the lower right in the RMLO image and from the upper right toward the lower left in the LMLO image. The outline component image is corrected according to the brightness weights for the given distance using the outline of the target component located at the top left or the top right corner (Figure 4).

#### 2.3.2. Edge Detection

**Figure 5:**
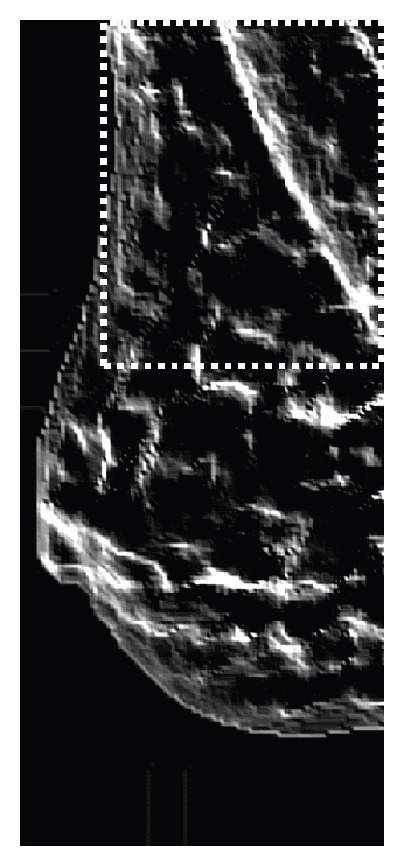
Detecting the candidate.

The breaststroke was removed and binarization was done in Otsu thresholding method in order to detect the outline candidates of the pectoral muscle. The labeling method was conducted on the binary image. The center point of the region was determined as the candidate contour of the pectoral muscle. The candidate contour exists within half of the height of the image including the breast region (Figure 5).

### 2.4. Pectoral Muscle Detection

#### 2.4.1. Create the Missing Lines

Hough transformation method was used for the detection of lines on the image generated by the edge detection. In this method, lines having similar angles are connected. The outline was detected in the LMLO view by connecting the lines having an angle in the range of 100°–170°. The outline was detected in the RMLO view by connecting the lines having an angle in the range of 280°–350°. This method detected candidate objects of the pectoral muscle that correspond to the length of the longest line after the connection.

#### 2.4.2. The Pectoral Muscle Detection Using RANSAC Fitting

**Figure 6:**
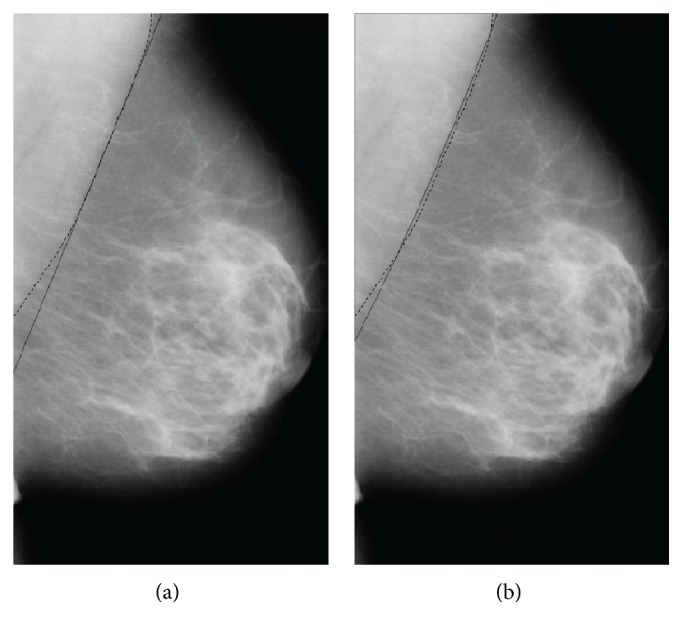
(a) Linear RANSAC method. (b) Nonlinear RANSAC method.

The pectoral muscle was interpolated by obtaining the information of the candidate objects after selecting the effective component of the quadratic curve fitting using the RANSAC algorithm (Figure 3(d)).  Figure 3(e) is the final image of the proposed method. The proposed method provides more accurate result than the linear RANSAC method by removing nonactive ingredients (Figure 6) [17].

The algorithm is as follows:

(1) Select *n* data of the data points randomly for interpolation.
(2) Initialize the number of iterations (*k*) to 1.
(3) Calculate the model after randomly importing *n* points from the set *X*.
(4) For tolerance *ε*, calculate the incoming data model and store the model in the tolerance.
(5) Increase the number of iterations (*k*) in steps of 1. If the selected maximum number of iterations (*K*) is such that *k* < *K*, then repeat the algorithm from step (3). If *k* is equal to *K*, then the model has the most consensus set in the interpolation models.

## 3. Results

**Figure 7:**
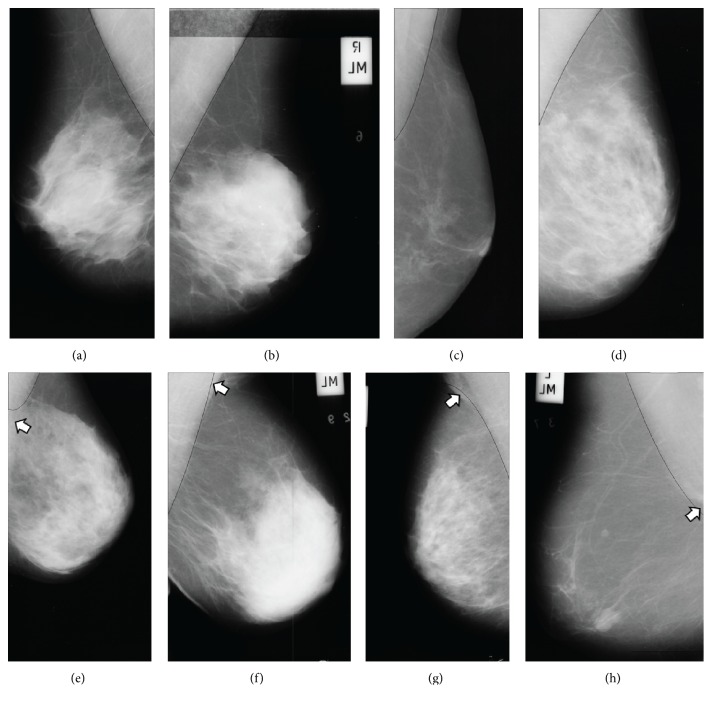
Detection results of mammographic image: (a–d) good detections and (e–h) acceptable and unacceptable detections.

The proposed system detected the pectoral muscle using the MIAS database. The database consists of the 322 images in the MLO view. The difference between the automatically and manually drawn regions was evaluated. If the concordance between the manually and automatically drawn images is more than 50% and less than 90%, the results were defined as “acceptable” (Figures 7(f)-7(g)), and if it is higher than 90% the results were defined as “good” (Figures 7(a)–7(d)). In addition, the results were defined as “unacceptable” if the concordance is lower than 50% (Figure 7(e); Table 1).

The evaluation result for the identifiable pectoral muscle region (“acceptable” and “good”) is as in Table 2.

The FP and FN were 4.51% and 5.68%, respectively. The accuracy was 92.2%. The two regions appeared visually correct when both the FP and FN differences were less than 5%. A little difference was observed in some regions visually when FP and FN were lower than 5% and 15%, respectively.

The mdb098 and mdb137 and mdb236 of images are barely observed. However, our method has been proposed under the assumption that all images are MLO in MIAS. Also these cases are included to category of “unacceptable”; it did not reflect on the accuracy, because these cases are not identified with naked eye.

## 4. Discussion

This study proposed a pectoral muscle detection method using the MIAS database. It used all the images in the MIAS database. The proposed method showed improved or similar results when compared with other studies. The detection accuracy of the proposed method (92.2%) was higher than that of Alam and Islam (90.3%) and Molinara et al. (89.1%). Particularly, the proposed method showed significantly improved detection ratio of the pectoral muscle region compared to the other studies when the FP and the FN rate differences were less than 5%. Molinara et al. also used the RANSAC method. This method has less detection accuracy because the shape of the outline is not always straight. By comparison, the proposed method showed high accuracy in some mammographic images when the outline was not a straight line. It determined the shape of a curve more accurately by applying the second equation. Alam and Islam detected the pectoral muscle using the *K*-means method. Furthermore, Alam and Islam detected the pectoral muscle region using the intensity value on the assumption that the pectoral muscle has a triangular shape. This detection method showed imperfect result along with the subjective visual region without any assessment of accuracy. In addition, a straight-line detection method such as the Hough transform and the watershed method were used.

Besides, we conducted test for images on FFDMs (66 images; Asan Medical Center, Korea). As a result, we obtained the result of the accuracy 95.86% because FFDMs have high resolution better than scanned film-based images. FFDMs are more popular in clinic practice; however, MIAS images are widely used as a common data to compare the performance with other methods (Table 3).

## 5. Conclusion

In many mammographic images, the pectoral muscle was detected more accurately because it resembles diagonal. However, it was difficult to detect the pectoral muscles when they are formed of curves. The proposed method showed higher accuracy in detecting curved shapes than the traditional methods. Folded skins sometimes also lead to strong edges on PM regions. Three approaches were used to solve the problems. At first, orthogonal kernel was used. This kernel was optimized to find blurred boundaries. Upon the images, PM edge was blurred and folded skin had sharp boundary. So the kernel could not strongly detect a folded skin, but PM regions. Secondly, distance weight was used. Folded skins were detected outside of the breast region, so the skins have lower distance weight than PM edge. PM regions were detected accurately because they have strong distance weight. At last, RANSAC was used. Folded skins and PM edges were detected together in general, so two edges were processed together in RANSAC fitting. In the result of cases, PM edges have longer edge than folded skin edges. These features were helpful to segment PM edges. Nevertheless, if the pectoral muscle has a complex shape, then detection rate was low. The detection accuracy of the contour component has a major effect on the detection of the candidate object. The proposed method will be tested to detect lesions using additional images. In addition, it is predicted that the proposed method will be complemented by using Garbor filter to obtain the orientation of the image. In the future, the result of proposed method is expected to be useful for the CAD systems in mammography.

## Figures and Tables

**Table 1:** Results of classification.

	Good	Acceptable	Unacceptable
Images	264	36	22

**Table 2:** Pectoral muscle detection performance.

Category	Percentage
FP	4.51 ± 6.53
FN	5.68 ± 8.57
FP < 5% and FN < 5%	56.5
5% < FP < 15%, 5% < FN < 15%	31.5
15% < FP, 15% < FN	12.0

**Table 3:** Results of comparison with other methods.

Methods	Images	Acc.	Unacc.
Kwok et al. [11]	322	83.6	16.4
Mustra and Grgic [16]	40	85.0	15.0
Raba et al. [9]	322	86.0	14.0
Molinara et al. [15]	55	89.1	10.9
Alam and Islam [10]	322	90.3	9.7
Our method	322	92.2	7.8
